# Stable use of radiotherapy in lymphoma patients over time – A comprehensive national overview of radiotherapy use in Sweden with focus on older patients

**DOI:** 10.1016/j.ctro.2024.100785

**Published:** 2024-04-21

**Authors:** Ingrid Glimelius, Sara Ekberg, Karin Ekström Smedby, Tove Wästerlid

**Affiliations:** a Clinical Epidemiology Division, Department of Medicine Solna, Karolinska Institutet, Stockholm, Sweden; b Department of Immunology, Genetics and Pathology, Cancer Precision Medicine, Uppsala University, Sweden; c Department of Hematology, Karolinska University Hospital, Stockholm, Sweden

**Keywords:** Radiotherapy, Lymphoma, Treatment, Survival, Elderly

## Abstract

**Background and purpose:**

The role of radiotherapy (RT) in lymphoma is constantly refined with the advent of novel treatments. However, RT is still an effective treatment and tolerability is high. Therefore, we aimed to describe the use of RT in primary treatment of lymphoma over calendar time, with a specific focus on older patients (age ≥ 70 years) with non-Hodgkin lymphoma (NHL) subtypes.

**Materials & Methods:**

All adult patients diagnosed with lymphoma from 2007 to 2018 in Sweden were included and followed for survival until end of 2020. Patient characteristics and relative survival (RS) were described for patients with NHL by subtype and RT use.

**Results:**

In the cohort of lymphoma patients aged ≥ 70 years (n = 12,698) 11 % received RT as part of primary treatment. No decline in use of RT over calendar period was seen. Use of RT as monotherapy was associated with stage I-II disease and older age among patients with stage III-IV disease. Patients with indolent lymphomas aged ≥ 70 years who were selected for treatment with RT as monotherapy with a dose of ≥ 20 Gy had 2-year RS rate of 100 % which remained similar at five years. For patients with DLBCL, RT as monotherapy with a dose of ≥ 20 Gy was mostly administered to patients aged ≥ 85 years with a 2-year RS rate of 68 %.

**Conclusion:**

The use of RT in first-line lymphoma treatment was stable over calendar time. RT monotherapy is associated with encouraging outcomes among patients with NHL aged ≥ 70 years who were selected to receive this.

## Highlights

• The use of RT in lymphoma has not decreased during the recent decade.
• Use of RT as monotherapy is most often applied to patients aged > 80 years or with limited-stage disease.
• Relative survival is encouraging for lymphoma patients selected for RT monotherapy.

## 1 Introduction

Lymphomas are clearly radiosensitive, but the role and use of radiotherapy (RT) in lymphoma treatment protocols are constantly refined with the introduction of novel drugs and immunotherapy. RT is associated with a risk of long-term side effects, especially following historical protocols with large extended RT fields [1], [2], [3], [4]. More advanced techniques that enable treatment of larger target volumes with lower radiation doses have reduced this risk [5], [6], [7]. Implementation of involved site radiation therapy (ISRT) has also contributed to decreased toxicity of RT [8], [9] as has novel RT modalities such as volumetric modulated arc therapy (VMAT) and proton radiotherapy [7]. Concomitantly, more effective and tolerable novel agents and chemoimmunotherapy (CIT) regimens are being developed, potentially leading to decreased use of RT for many lymphoma subtypes. RT is regularly applied for a range of indications in lymphoma, but trends over time and survival outcomes in the modern era, particularly among older patients, is not known on a population-based level.

In Diffuse Large B-cell lymphoma (DLBCL), 3 cycles of rituximab- cyclophosphamide, doxorubicin, vincristine, and prednisone (R-CHOP) followed by RT has been a standard treatment option for patients with stage I disease [10]. Further, RT is recommended as consolidative treatment for patients with incomplete remission after induction chemotherapy and historically to improve outcome for patients with skeletal manifestation and/or bulky disease [11], [12], [13]. Similarly to patients with DLBCL, the use of 3 cycles of CHOP + RT (40 Gray (Gy), 20 fractions) for patients with limited-stage T-cell lymphomas (TCL) is considered a standard treatment option [14], [15]. Also in mantle cell lymphoma (MCL), RT is considered standard of care for stage I − limited stage II disease [16], [17].

For indolent lymphomas such as follicular lymphoma (FL) and marginal zone lymphoma (MZL), RT constitutes a potentially curative treatment option for patients with stage I and limited stage II disease [18]. Standard dose is 24 Gy (2Gyx12) as 24 Gy demonstrated equal efficacy to 40 Gy (2 Gy x20) in a randomised study for indolent lymphomas [19].

RT can also function as a tolerable palliative treatment and achieve symptom relief and local disease control. Here, 4 Gy(2Gyx2) is considered a feasible option to relieve symptoms and achieve local control also in more advanced stages of lymphoma. This may become more important as the number of older patients with lymphoma is expected to grow with increased life expectancy [20], [21]. Also, potential long-term side effects of RT do not pose a problem among the elderly. However, its use and role in this setting has not been described.

With novel targeted therapies currently developing at a quick pace the use of RT as curative, consolidative and/or palliative treatment does not get as much focus. Recent data on the use and efficacy of RT in lymphomas is lacking. Therefore, we aimed to describe the use of RT among lymphoma patients overall and by calendar time, with a specific focus on patient characteristics and relative survival of older lymphoma patients (age ≥ 70 years) with NHL subtypes.

## 2 Materials & Methods

### 2.1 Study population

All adult patients diagnosed with lymphoma from 1st January 2007 to 31st of December 2018 and registered in the Swedish lymphoma register (SLR) were included. The SLR was established in 2000 by the Swedish Lymphoma Group and contains detailed clinical data on disease characteristics, prognostic indices, treatment (added 2007), response and outcomes among all adult patients diagnosed with lymphomas in Sweden. Compared to the mandatory Swedish Cancer Register, the coverage of the SLR is > 95 % [23]. RT is registered as a separate treatment variable as is RT dose (Gy) but the register does not contain information regarding RT fields. Administration of RT as monotherapy was divided into two groups, RT doses ≥ 20 Gy (compatible with curative intent for indolent lymphomas) and RT doses < 20 Gy, compatible with palliative intent, with the vast majority receiving 2x2Gy for indolent lymphomas and 2x4Gy for aggressive lymphomas.

To describe the use of RT overall, all patients registered in the SLR were included and stratified by subtype. The proportion of patients who received RT, either as monotherapy or in combination with chemoimmunotherapy, was plotted over calendar period. For the main analysis of patient characteristics and relative survival (RS) we focused on patients with DLBCL, FL, MZL, MCL, and TCL for whom the use of RT is less well described. Further, only patients aged ≥ 70 years were included as the use of RT might be especially useful for older patients with lymphoma.

### 2.2 Statistical methods

RS was used as a measure of net survival and was estimated as the ratio of the observed all-cause survival to the expected survival in an age-, sex-, country- and calendar-period-matched population (assumed lymphoma-free). The Ederer II method was used to calculate expected survival in the matched Swedish general populations using data obtained via the Human Mortality Database (www.mortality.org). RS analyses were performed by subtype and stratified by treatment type and presented in cumulative RS graphs and as 5-year RS estimates with 95 % confidence intervals (CI). Follow-up was started at the end of treatment to avoid immortal time bias.

The study was approved by the Ethical Review Board in Stockholm, Sweden (2015–202831).

## 3 Results

### 3.1 Use of RT overall

In total, 25,382 patients were registered with a lymphoma diagnosis in the SLR 2007–2018. Of these, 3,393 (13 %) received RT as part of their primary treatment. Among patients aged ≥ 70 years (n = 12,698), 1,376 (11 %) received RT. When excluding HL (not further described in the present study) from Table 1 the proportion of patients who received RT was 12 % and 11 % among patients aged </≥70 years, respectively. The proportion of patients who received RT by subtype is presented in Table 1. The use of RT was uncommon for patients with lymphoplasmacytic lymphoma and small lymphocytic lymphoma (1 % and 4 %, respectively) wherefore these subtypes are not further described in this study.

**Table 1:** Distribution of lymphoma subtypes among all diagnosed (n = 25,382) and all radiotherapy-treated (n = 3,393) lymphoma patients diagnosed 2007–2018 in Sweden. Patients aged ≥ 70 years reported in the column to the right.

	All ages		Age ≥ 70 years	
Lymphoma subtype	Total N (col%)	RT N (row%)	Total N (col%)	RT N (row%)
**Total**	25,382 (1 0 0)	3,393 (13.4)	12,698 (1 0 0)	1,376 (10.8)
**Aggressive subtypes**	13,794 (54.4)	2,150 (15.6)	6,524 (51.4)	867 (13.3)
**DLBCL**	7,831 (30.9)	1,211 (15.5)	4,174 (32.9)	639 (15.3)
**T-cell lymphoma**	1,795 (7.1)	197 (11.0)	823 (6.5)	81 (9.8)
**Hodgkin lymphoma**	2,066 (8.1)	596 (28.9)	402 (3.2)	75 (18.7)
**Mantle cell lymphoma**	1,422 (5.6)	88 (6.2)	806 (6.4)	42 (5.2)
**Aggressive, not specified**	680 (2.7)	58 (8.5)	319 (2.5)	30 (9.4)
**Indolent subtypes**	8,686 (34.2)	1,118 (12.9)	4,345 (34.2)	430 (9.9)
**Follicular lymphoma**	3,309 (13.0)	601 (18.2)	1,367 (10.8)	220 (16.1)
Lymphoplasmacytic lymphoma*	1,482 (5.8)	17 (1.2)	927 (7.3)	12 (1.3)
**Marginal zone lymphoma**	1,601 (6.3)	324 (20.2)	787 (6.2)	131 (16.7)
**SLL**	685 (2.7)	26 (3.8)	431 (3.4)	16 (3.7)
**NLPHL**	134 (0.5)	71 (53.0)	15 (0.1)	4 (26.7)
**Indolent, not specified**	1,475 (5.8)	79 (5.4)	818 (6.4)	47 (5.8)
Other/Missing subtype**	2,902 (11.4)	125 (4.3)	1,829 (14.4)	79 (4.3)

*including Mb Waldenström, **Not specified other: n = 1045, Missing subtype data: n = 1857. DLBCL = Diffuse Large B-cell Lymphoma, SLL = Small Lymphocytic Lymphoma, NLPHL = Nodular Lymphocyte Predominant Hodgkin Lymphoma, RT = Radiotherapy, N = Number of patients.

### 3.2 Use of RT by calendar year

**Fig. 1:**
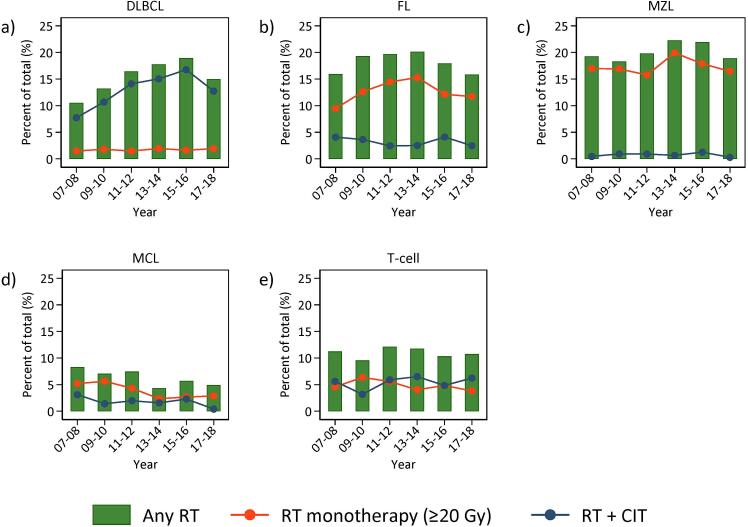
Proportions of patients treated with radiotherapy (RT) as part of their primary treatment by calendar period, subtype and mode of use (any RT, RT monotherapy ≥ 20 Gy, or RT in combination with chemoimmunotherapy (CIT)). Abbreviations: DLBCL = Diffuse Large B-cell Lymphoma, FL = Follicular Lymphoma, MZL = Marginal Zone Lymphoma, MCL = Mantle Cell Lymphoma, chemo = chemotherapy, RT = Radiotherapy, Gy = Gray.

The use of RT overall was plotted over calendar period, subtype (DLBCL, FL, MZL, MCL, TCL) and presented overall and by use of RT as monotherapy or in combination with CIT (Fig. 1). With some variation by calendar years, the use of RT remained stable over time for all subtypes.

### 3.3 Use of RT in patients with DLBCL aged ≥ 70 years

Among 4,174 patients with DLBCL aged ≥ 70 years at diagnosis, 639 (15 %) received RT (Table 2A). Of these, 114 (18 %) received RT as monotherapy with a dose ≥ 20 Gy, 57 (9 %) received RT as monotherapy with a dose < 20 Gy and the remainder (n = 468, 73 %) received RT + CIT. Among patients managed with RT + CIT, 50 % (n = 232) did not receive full chemotherapy with 6 cycles of which 47 % (n = 108) received R-CHOPx3 + RT as standard treatment for low-stage disease. The majority of patients who received RT monotherapy with dose ≥ 20 Gy (median 30 Gy) presented with stage I-II disease (72 %). Further, median age was higher (85 years) and there was a higher proportion of patients with extranodal disease (47 %) in this cohort compared to patients who received CIT. As expected, median age was high (89 years) and the proportion of patients with poor performance status (PS) was large (PS 2–4: 46 %) in the cohort of patients who received RT monotherapy with a dose < 20 Gy (median 4 Gy). Among patients who received RT + CIT, there was a larger proportion of patients with extranodal disease (28 %) compared to patients who received CIT only (15 %). Interestingly, female patients predominated in the cohort of patients who received RT monotherapy, 60 % and 65 % in dose ≥ 20 Gy and < 20 Gy cohorts, respectively.

**Table 2A:** Clinical characteristics of patients with diffuse large B cell lymphoma (DLBCL) aged ≥ 70 years, stratified by treatment type with or without radiotherapy (RT) and by RT dose.

	**RT**						**No RT**	
	**RT monotherapy** **≥20 Gy**		**RT monotherapy** **<20 Gy**		**RT** **+ CIT**			
**Total (row %)**	114	(2.7)	57	(1.4)	468	(11.2)	3535	(84.7)
**Median RT dose, Gy (IQ range)**	30	(24–36)	4	(4–8)	30	(26–30)		
**Systemic treatment type**								
R-CHOP/R-CHOP-like	−	−	−	−	399	(85.3)	2336	(66.1)
Other	−	−	−	−	69	(14.7)	298	(8.4)
No R-Chemo	114	(1 0 0)	57	(1 0 0)	NA		901	(25.5)

**Number of chemotherapy cycles**								
<6	−	−	−	−	232	(49.9)	758	(29.0)
≥6	−	−	−	−	233	(50.1)	1858	(71.0)
**Median Age (IQ range)**	85	(81–89)	89	(86–91)	78	(73–83)	78	(74–83)

**Age group**								
70–84 years	49	(43)	10	(17.5)	394	(84.2)	2856	(80.8)
85+	65	(57)	47	(82.5)	74	(15.8)	679	(19.2)

**Sex**								
Male	46	(40.4)	20	(35.1)	262	(56.0)	1897	(53.7)
Female	68	(59.6)	37	(64.9)	206	(44.0)	1638	(46.3)

**Calendar period**								
2007–2013	61	(53.5)	39	(68.4)	219	(46.8)	1945	(55.0)
2014–2018	53	(46.5)	18	(31.6)	249	(53.2)	1590	(45.0)

**Stage (Ann Arbor)**								
I	62	(54.4)	25	(43.9)	179	(38.2)	609	(17.2)
II	20	(17.5)	11	(19.3)	99	(21.2)	753	(21.3)
III-IV	20	(17.5)	14	(24.6)	181	(38.7)	1898	(53.7)
Missing	12	(10.5)	7	(12.3)	9	(1.9)	275	(7.8)

**Performance status**								
ECOG 0–1	77	(67.5)	31	(54.4)	380	(81.2)	2291	(64.8)
ECOG 2–4	34	(29.8)	26	(45.6)	78	(16.7)	1137	(32.2)
Missing	3	(2.6)	0	(0.0)	10	(2.1)	107	(3.0)

**Extranodal disease**								
No	49	(43.0)	36	(63.2)	330	(70.5)	2718	(76.9)
Yes	53	(46.5)	14	(24.6)	129	(27.6)	542	(15.3)
Missing	12	(10.5)	7	(12.3)	9	(1.9)	275	(7.8)

**Bulky disease**								
No	104	(91.2)	46	(80.7)	368	(78.6)	2682	(75.9)
Yes	5	(4.4)	7	(12.3)	97	(20.7)	644	(18.2)
Missing	5	(4.4)	4	(7.0)	3	(0.6)	209	(5.9)

Gy = Gray, CIT = immunochemotherapy.

**Fig. 2:**
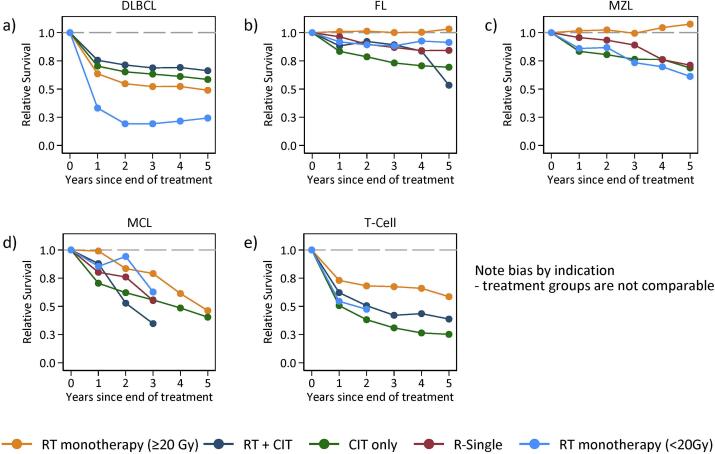
Relative Survival (RS) by lymphoma subtype and treatment type including radiotherapy (RT) only (≥20 Gy or < 20 Gy), chemoimmunotherapy (CIT) with or without RT or rituximab (R) single.

For DLBCL, 2- & 5-year RS rate for patients managed with RT monotherapy with doses ≥ 20 Gy was 68 % and 67 % respectively, compared to patients treated with CIT who had a 2- & 5-year RS of 77 % and 70 %, respectively. Patients with DLBCL who received RT monotherapy with doses < 20 Gy had 2-year RS of 29 % (Fig. 2, Table 3).

### 3.4 Use of RT in patients with FL and MZL aged ≥ 70 years

Among 1,367 patients with FL aged ≥ 70 years, 220 (16 %) received RT (Table 2B). The majority received RT monotherapy (181/220, 82 %). Of these, 136 (75 %) patients received RT with a curative dose ≥ 20 Gy (median 24 Gy), and as expected, the majority of these patients had stage I-II disease (91 %). Median age in this cohort was similar to non-RT treated patients (76 years). Median age was higher (82 years) and stage distribution was more varied among patients who received RT monotherapy with a dose < 20 Gy (median 4 Gy). Again, females predominated in RT monotherapy cohorts (56 % and 67 % in dose ≥ 20 Gy and < 20 Gy cohorts, respectively).

**Table 2B:** Clinical characteristics of patients with follicular lymphoma (FL) aged ≥ 70 years, stratified by treatment type with or without radiotherapy (RT), and by RT dose.

	**RT**						**No RT**	
	**RT monotherapy ≥20 Gy**		**RT monotherapy** **<20 Gy**		**RT** **+ CIT**			
**Total, row %**	136	(10)	45	(3.3)	39	(2.9)	1147	(83.9)
**Median Gy (IQ range)**	24	(24–30)	4	(4–8)	30	(24–30)		
**Systemic treatment type**								
BR	NA		NA		12	(30.8)	141	(12.3)
R-CHOP/R-CHOP-like	NA		NA		22	(56.4)	245	(21.4)
Rituximab only	NA		NA		NA		176	(15.3)
Other	NA		NA		5	(12.8)	142	(12.4)
No R-Chemo	136	(1 0 0)	45	(1 0 0)	0	(0.0)	443	(38.6)

**Number of cycles**								
<6	NA		NA		20	(52.6)	196	(38.2)
≥6	NA		NA		18	(47.4)	317	(61.8)
**Median Age (IQ range)**	76	(72–80)	82	(75–87)	76	(72–81)	77	(73–81)

**Age group (years)**								
70–84	121	(89.0)	28	(62.2)	36	(92.3)	993	(86.6)
85+	15	(11.0)	17	(37.8)	3	(7.7)	154	(13.4)

**Sex**								
Male	60	(44.1)	15	(33.3)	15	(38.5)	545	(47.5)
Female	76	(55.9)	30	(66.7)	24	(61.5)	602	(52.5)

**Calendar period**								
2007–2013	76	(55.9)	28	(62.2)	15	(38.5)	595	(51.9)
2014–2018	60	(44.1)	17	(37.8)	24	(61.5)	552	(48.1)

**Stage (Ann Arbor)**								
I	110	(80.9)	15	(33.3)	12	(30.8)	183	(16.0)
II	15	(11.0)	6	(13.3)	9	(23.1)	218	(19.0)
III-IV	10	(7.4)	17	(37.8)	17	(43.6)	682	(59.5)
Missing	1	(0.7)	7	(15.6)	1	(2.6)	64	(5.6)

**Performance status**								
ECOG 0–1	129	(94.9)	42	(93.3)	36	(92.3)	997	(86.9)
ECOG 2–4	3	(2.2)	3	(6.7)	3	(7.7)	124	(10.8)
Missing	4	(2.9)	0	(0.0)	0	(0.0)	26	(2.3)

**Extranodal disease**								
No	117	(86.0)	34	(75.6)	35	(89.7)	1,032	(90.0)
Yes	18	(13.2)	4	(8.9)	3	(7.7)	51	(4.4)
Missing	1	(0.7)	7	(15.6)	1	(2.6)	64	(5.6)

**Bulky disease**								
No	131	(96.3)	41	(91.1)	28	(71.8)	905	(78.9)
Yes	5	(3.7)	2	(4.4)	11	(28.2)	198	(17.3)
Missing	0	(0.0)	2	(4.4)	0	(0.0)	44	(3.8)

Gy = Gray, CIT=chemoimmunotherapy

Of 781 MZL patients, 131 (%) received RT, mainly as monotherapy (Table 2C). The majority (80 %) received RT doses ≥ 20 Gy (median 24 Gy) and 20 % RT dose < 20 Gy (median 4 Gy). The majority of patients managed with RT with curative intent dosing had stage I-II disease (90 %). Extranodal disease was also common (62 %) in this group. Female patients predominated in the RT monotherapy cohort with doses ≥ 20 Gy cohort (63 % compared to 54 % females among non-RT treated patients).

**Table 2C:** Clinical characteristics of patients with marginal zone lymphoma (MZL) aged ≥ 70 years, stratified by treatment type with or without radiotherapy (RT), and RT dose.

	**RT**				**No RT**	
	**RT monotherapy** **≥20 Gy**		**RT monotherapy** **<20 Gy**			
**Total (row %)**	100	(12.7)	25	(3.2)	656	(83.4)
**Median Gy (IQ range)**	24	(24–30)	4	(4–4)	NA	
**Systemic treatment type**						
BR	−		−		52	(7.9)
R-CHOP/R-CHOP-like	−		−		28	(4.3)
Rituximab only			−		113	(17.2)
Other	−		−		85	(13.0)
No R-Chemo	100	(1 0 0)	25	(1 0 0)	378	(57.6)

**Number of cycles**						
<6	−		−		85	(54.1)
≥6	−		−		72	(45.9)
**Median Age (IQ range)**	77	(73–81)	80	(74–85)	77	(73–82)

**Age group (years)**						
70–84	86	(86.0)	18	(72.0)	566	(86.3)
85+	14	(14.0)	7	(28.0)	90	(13.7)
**Sex**						
Male	37	(37.0)	15	(60.0)	300	(45.7)
Female	63	(63.0)	10	(40.0)	356	(54.3)

**Calendar period**						
2007–2013	45	(45.0)	9	(36.0)	323	(49.2)
2014–2018	55	(55.0)	16	(64.0)	333	(50.8)

**Stage (Ann Arbor)**						
I	82	(82.0)	8	(32.0)	173	(26.4)
II	8	(8.0)	2	(8.0)	56	(8.5)
III-IV	7	(7.0)	12	(48.0)	381	(58.1)
Missing	3	(3.0)	3	(12.0)	46	(7.0)

**Performance status**						
ECOG 0–1	97	(97.0)	21	(84.0)	574	(87.5)
ECOG 2–4	3	(3.0)	2	(8.0)	55	(8.4)
Missing	0	(0.0)	2	(8.0)	27	(4.1)

**Extranodal disease**						
No	35	(35.0)	15	(60.0)	452	(68.9)
Yes	62	(62.0)	7	(28.0)	158	(24.1)
Missing	3	(3.0)	3	(12.0)	46	(7.0)

**Bulky disease**						
No	96	(96.0)	23	(92.0)	595	(90.7)
Yes	3	(3.0)	1	(4.0)	25	(3.8)
Missing	1	(1.0)	1	(4.0)	36	(5.5)

Gy = Gray, *in addition, 6 individuals (0.8 %) received RT in combination with chemoimmunotherapy.

Patients aged ≥ 70 years with FL or MZL who received RT monotherapy with a dose of ≥ 20 Gy had 2-year RS rates of 100 % and 99 %, respectively. Patients selected for RT monotherapy with palliative dose < 20 Gy had 2-year RS-rates of 95 % for FL and 93 % for MZL. Rates were similar also for 5-year RS (Fig. 2, Table 3).

### 3.6 Use of RT in patients with MCL aged ≥ 70 years

Only 42 of 806 (5 %) patients with MCL aged ≥ 70 years received RT (Table 2D). Median age was higher in all RT groups (81, 86, and 83 years, respectively) compared to patients managed with CIT only (78 years). Stage I-II disease was more common in all RT groups. Males predominated in the cohort of MCL patients who received RT as monotherapy with a dose of ≥ 20 Gy (77 % males), whereas the proportion of females was larger in the RT monotherapy < 20 Gy cohort and RT + CIT cohort (78 % and 46 % females, respectively). For patients with MCL managed with RT monotherapy with RT doses ≥ 20 Gy, 2-year RS was 80 % (Fig. 2, Table 3).

**Table 2D:** Clinical characteristics of patients with mantle cell lymphoma (MCL) aged ≥ 70 years, stratified by treatment type with or without radiotherapy (RT), and RT dose.

	**RT**						**No RT**	
	**RT monotherapy ≥ 20 Gy**		**RT monotherapy** **<20 Gy**		**RT + CIT**			
**Total (row %)**	22	(2.7)	9	(1.1)	11	(1.4)	764	(94.8)
**Median Gy (IQ range)**	30	(30–30)	4	(4–8)	30	(8–30)	NA	

**Systemic treatment type**								
BR	−		−		2	(18.2)	284	(37.2)
R-CHOP/R-CHOP-like	−		−		4	(36.4)	127	(16.6)
Rituximab only	−		−		−		12	(1.6)
Other	−		−		5	(45.5)	138	(18.1)
No R-Chemo	22	(1 0 0)	9	(1 0 0)	0	(0)	203	(26.6)

**Number of cycles**								
<6	−		−		7	(63.6)	237	(44.2)
≥6	−		−		4	(36.4)	299	(55.8)
**Median Age (IQ range)**	81	(76–87)	86	(83–87)	83	(74–87)	78	(74–82)

**Age group (years)**								
70–84	15	(68.2)	4	(44.4)	8	(72.7)	633	(82.9)
85+	7	(31.8)	5	(55.6)	3	(27.3)	131	(17.1)

**Sex**								
Male	17	(77.3)	2	(22.2)	6	(54.5)	541	(70.8)
Female	5	(22.7)	7	(77.8)	5	(45.5)	223	(29.2)

**Calendar period**								
2007–2013	13	(59.1)	3	(33.3)	7	(63.6)	410	(53.7)
2014–2018	9	(40.9)	6	(66.7)	4	(36.4)	354	(46.3)

**Stage (Ann Arbor)**								
I	8	(36.4)	2	(22.2)	1	(9.1)	34	(4.5)
II	6	(27.3)	4	(44.4)	6	(54.5)	62	(8.1)
III-IV	7	(31.8)	3	(33.3)	4	(36.4)	619	(81.0)
Missing	1	(4.5)	0	(0)	0	(0)	49	(6.4)

**Performance status**								
ECOG 0–1	18	(81.8)	7	(77.8)	11	(1 0 0)	585	(76.6)
ECOG 2–4	3	(13.6)	2	(22.2)	0	(0)	163	(21.3)
Missing	1	(4.5)	0	(0)	0	(0)	16	(2.1)

**Extranodal disease**								
No	17	(77.3)	7	(77.8)	11	(1 0 0)	688	(90.1)
Yes	4	(18.2)	2	(22.2)	0	(0)	27	(3.5)
Missing	1	(4.5)	0	(0.0)	0	(0)	49	(6.4)

**Bulky disease**								
No	22	(1 0 0)	8	(88.9)	9	(81.8)	642	(84.0)
Yes	0	(0)	1	(11.1)	2	(18.2)	70	(9.2)
Missing	0	(0)	0	(0)	0	(0)	52	(6.8)

Gy = Gray CIT = chemoimmunotherapy.

### 3.7 Use of RT in patients with TCL aged ≥ 70 years

Overall, 81 of 823 patients with TCL received RT (10 %) (Table 2E). Of these, the majority (53 %) received RT monotherapy with dose ≥ 20 Gy (median 30 Gy). Median age was highest in the RT monotherapy groups (82 and 80 years, respectively) and stage I-II disease was more common in both RT groups, as was presence of extranodal disease (63 % and 50 %, respectively). For patients with TCL who were managed with RT monotherapy with RT dose ≥ 20 Gy, 2-year RS was 73 % compared to 53 % and 53 % with RT + CIT and CIT (Fig. 2, Table 3).

**Table 2E:** Clinical characteristics of patients with T-cell lymphoma (TCL) aged ≥ 70 years, stratified by treatment type with or without radiotherapy (RT), and RT dose.

	RT						No RT	
	RT monotherapy ≥ 20 Gy		RT monotherapy <20 Gy		RT + CIT			
**Total (row %)**	43	(5.2)	10	(1.2)	28	(3.4)	742	(90.2)
**Median Gy (IQ range)**	30	(25–40)	8	(8–16)	30	(18–40)	NA	
**Systemic treatment type**								
BR	−		−		0	(0)	3	(0.4)
R-CHOP/R-CHOP-like	−		−		20	(71.4)	274	(36.9)
Other	−		−		8	(28.6)	79	(10.6)
No R-Chemo	43	(1 0 0)	10	(1 0 0)	0	(0)	386	(52)

**Number of cycles**								
<6	−		−		16	(59.3)	145	(43.3)
≥6	−		−		11	(40.7)	190	(56.7)
**Median Age (IQ range)**	82	(74–87)	80	(77–86)	75	(73–80)	77	(73–82)

**Age group (years)**								
70–84	27	(62.8)	7	(70)	24	(85.7)	617	(83.2)
85+	16	(37.2)	3	(30)	4	(14.3)	125	(16.8)

**Sex**								
Male	27	(62.8)	7	(70)	18	(64.3)	442	(59.6)
Female	16	(37.2)	3	(30)	10	(35.7)	300	(40.4)

**Calendar period**								
2007–2013	30	(69.8)	4	(40)	12	(42.9)	397	(53.5)
2014–2018	13	(30.2)	6	(60)	16	(57.1)	345	(46.5)

**Stage (Ann Arbor)**								
I	23	(53.5)	5	(50)	11	(39.3)	111	(15)
II	7	(16.3)	0	(0)	6	(21.4)	59	(8)
III-IV	6	(14.0)	2	(20)	11	(39.3)	413	(55.7)
Missing	7	(16.3)	3	(30)	0	(0)	159	(21.4)

**Performance status**								
ECOG 0–1	39	(90.7)	9	(90)	24	(85.7)	489	(65.9)
ECOG 2–4	1	(2.3)	1	(10)	4	(14.3)	209	(28.2)
Missing	3	(7.0)	0	(0)	0	(0)	44	(5.9)

**Extranodal disease**								
No	9	(20.9)	2	(20)	18	(64.3)	478	(64.4)
Yes	27	(62.8)	5	(50)	10	(35.7)	105	(14.2)
Missing	7	(16.3)	3	(30)	0	(0)	159	(21.4)

**Bulky disease**								
No	38	(88.4)	10	(1 0 0)	27	(96.4)	622	(83.8)
Yes	1	(2.3)	0	(0)	1	(3.6)	34	(4.6)
Missing	4	(9.3)	0	(0)	0	(0)	86	(11.6)

Gy = Gray, CIT = chemoimmunotherapy.

**Table 3:** Relative survival estimates at 2- and 5-years with 95% confidence intervals stratified by subtype and treatment type. Data not presented if less than 5 individuals in each strata.

Subtype/Treatment	2-year RS (95 % CI)	5-year RS (95 % CI)
**DLBCL**		
Single RT (>=20 Gy)	0.68 (0.53, 0.81)	0.67 (0.46, 0.88)
RT + R-CHEMO	0.76 (0.71, 0.81)	0.70 (0.63, 0.77)
R-CHEMO (−RT)	0.77 (0.75, 0.79)	0.70 (0.67, 0.73)
R-Single	NA	NA
Single RT (<20 Gy)	0.29 (0.13, 0.49)	0.34 (0.13, 0.66)

**FL**		
Single RT (>=20 Gy)	1.00 (0.94, 1.04)	1.03 (0.92, 1.11)
RT + R-CHEMO	0.94 (0.77, 1.02)	0.65 (0.38, 0.87)
R-CHEMO (−RT)	0.84 (0.80, 0.88)	0.73 (0.66, 0.78)
R-Single	0.95 (0.88, 1.00)	0.89 (0.78, 0.99)
Single RT (<20 Gy)	0.94 (0.76, 1.05)	0.92 (0.67, 1.13)

**MZL**		
Single RT (>=20 Gy)	0.99 (0.90, 1.04)	1.05 (0.89, 1.16)
RT + R-CHEMO	NA	NA
R-CHEMO (−RT)	0.89 (0.81, 0.95)	0.76 (0.64, 0.86)
R-Single	0.93 (0.83, 0.99)	0.76 (0.60, 0.90)
Single RT (<20 Gy)	1.00 (0.72, 1.11)	0.79 (0.37, 1.12)

**MCL**		
Single RT (>=20 Gy)	0.80 (0.49, 1.00)	0.54 (0.21, 0.91)
RT + R-CHEMO	0.53 (0.19. 0.83)	NA
R-CHEMO (−RT)	0.73 (0.68, 0.78)	0.51 (0.45, 0.58)
R-Single	0.91 (0.45, 1.09)	NA
Single RT (<20 Gy)	1.06 (0.47, 1.19)	NA

**TCL**		
Single RT (>=20 Gy)	0.73 (0.52, 0.89)	0.66 (0.41, 0.90)
RT + R-CHEMO	0.53 (0.29, 0.73)	0.44 (0.20, 0.69)
R-CHEMO (−RT)	0.53 (0.46, 0.59)	0.34 (0.26, 0.42)
R-Single	NA	NA
Single RT (<20 Gy)	0.67 (0.25, 0.97)	NA

NA = Not available, RS = Relative Survival, DLBCL = Diffuse Large B-cell Lymphoma, FL = Follicular Lymphoma, MZL = Marginal Zone Lymphoma, MCL = Mantle Cell Lymphoma, TCL = T cell lymphoma, chemo = chemotherapy, RT = Radiotherapy, Gy = Gray, R = Rituximab.

### 3.8 Use of RT and patient characteristics by sex

When comparing base-line characteristics by sex and subtype, female patients were significantly older in the DLBCL and TCL cohorts and there was a trend for higher age among female patients for all other lymphoma subtypes (data not shown). In addition, female patients with DLBCL more often presented with stage I disease (25 % among females, 20 % of males, p = 0.01). Apart from this, there were no significant sex differences in base-line characteristics among any lymphoma subtypes.

## 4 Discussion

We report nationwide, population-based data on the use of RT as part of primary treatment for lymphoma in Sweden with a specific focus on patients aged ≥ 70 years and NHL subtypes. We demonstrate that the use of RT has remained relatively constant during the recent decade. Further, we show encouraging RS rates for patients aged ≥ 70 years who were selected for RT, *i.e.* especially for patients with low-stage indolent subtypes managed with RT only with curative intent but also among patients with aggressive lymphoma selected to receive ≥ 20 Gy.

This unique population-based study shows an unbiased reflection of the use of RT in lymphoma patients overall. The stable use over calendar time is reassuring since RT in selected patients showed encouraging RS rates. Regarding the use of RT as monotherapy this is, as expected, mostly administered to patients with low-stage disease, especially among indolent subtypes where this may be a curative treatment option. Extranodal involvement was more common among indolent subtypes managed with curative intent RT, consistent with guidelines for the treatment of both FL and MZL [24], [25]. Among patients with DLBCL, presence of extranodal disease predisposed for treatment with RT + CIT in the present study. This is in accordance with previous guidelines [26], but is no longer routinely recommended as the benefit of RT in this setting are conflicting [12], [26].

For all subtypes except TCL we observe a female predominance in the RT monotherapy cohorts. Potentially, this is explained by the longer life expectancy of females as RT monotherapy was more commonly administered to the oldest patients. Indeed, females with DLBCL and TCL were significantly older than males with DLBCL and TCL and there was a trend for higher age among females for all subtypes. Another potential explanation may be that female patients seek health care earlier and thus more often present with low-stage disease [27]. However, apart from age and a larger proportion of patients with stage I disease among female DLBCL patients no differences in base-line characteristics by sex and subtype were found. The use of RT by sex has rarely been described in population-based series on lymphoma patients.

It is important to note that the RS curves presented in this study should not be interpreted as a comparison between treatment regimens as bias by indication is unavoidable. As evident by the patient characteristics for patients managed with RT monotherapy (for example more often stage I-II disease, potentially curable with RT in FL and MZL) the differences in survival is likely largely driven by these underlying differences in patient characteristics that made them eligible for this specific treatment in the first place. Regardless, the very encouraging RS rates observed for patients with FL and MZL who were selected for treatment with RT monotherapy supports that this is a feasible treatment option with limited toxicity for older patients. That this is a selected group of patients is indicated by the better survival among patients with FL and MZL managed with RT monotherapy with curative dose compared to the age, sex and year matched general population. *I.e*., to be evaluated for a potentially asymptomatic indolent lymphoma, you are probably healthier than many other individuals aged ≥ 70 years. It is interesting that we did not observe large differences with regard to RT dose </≥ 20 Gy among patients with FL and MZL who received RT monotherapy. Again, this may be due to bias by indication but also indicate that the response obtained with low-dose RT has sufficient duration in this older cohort. This is in contrast to a randomised study on indolent lymphomas where longer remissions were observed with 24 Gy compared to 4 Gy [28].

For patients with DLBCL, MCL and TCL who received RT monotherapy with a dose ≥ 20 Gy we also observe unexpectedly high RS rates. Again, this indicates a clear selection of patients to RT with the majority of high-risk patients likely included in the CIT cohorts. Indeed, patients selected for RT predominantly had stage I-II disease and were thus more suitable to receive RT monotherapy even though this is not considered curative for aggressive lymphoma subtypes. The majority of patients with aggressive subtypes who received RT monotherapy were aged ≥ 80 years (≥85 years for DLBCL). Overall, these data indicate that in selected cases, RT monotherapy constitutes an efficacious and cost-effective treatment option for the oldest old lymphoma patients who may not be eligible for chemotherapy.

As expected, RS rates were lower for patients with DLBCL, MCL and TCL who were managed with RT monotherapy with RT dose < 20 Gy, likely administered solely as palliative symptom control. In this setting, survival rates are of less interest. Still, although numbers of patients are small, it is interesting to note that there is a proportion of patients in this older cohort aged ≥ 70 years with DLBCL, MCL and TCL who appear to have a long-term benefit of RT monotherapy with a dose of < 20 Gy, as indicated by plateauing curves. Also, that RT is a feasible palliative treatment option that can provide relief of symptoms has been shown in previous studies [22], [29]. In a small phase-two study of 25 DLBCL patients treated with low-dose RT (2Gyx2), an overall response rate of 70 % was observed, along with symptom relief in 60 % of patients [22]. In addition, a small proportion of DLBCL patients who received RT only with RT dose ≥ 20 Gy presented with stage III-IV disease. Thus, the treatment intent for these patients was likely also purely palliative, despite a higher RT dose.

The encouraging RS seen in selected lymphoma patients receiving RT, and the reduced risk of side effects and second malignancies with modern RT concepts, should encourage future clinical trials and continued inclusion of RT as a treatment option in national and international guidelines [30]. Newer studies also show the safety of combining RT with novel targeted drugs and immunotherapy [31], [32].

A limitation in the present study is the lack of data regarding the use of RT in later lines of treatment, as only primary treatment is comprehensively registered in the SLR. It is likely that RT is applied to a larger number of patients when also including the relapse setting. Further, this study is solely descriptive and RS-rates should not be interpreted as comparable between treatment regimens due to inevitable prominent bias by indication. An advantage of presenting RS rates is the fact that all excess mortality caused by the lymphoma is captured, including potential treatment toxicity.

## 5 Conclusion

To conclude, we demonstrate that the use of RT has not decreased in the recent decade. Further, we show that RT still constitutes an effective and tolerable treatment modality for selected patients, especially patients with low-stage disease. Thus, RT as a treatment modality should not be forgotten, especially among older patients who may not tolerate more intensive treatment.

## Role of funding source

This study was supported by the Swedish Cancer Society, 22 2167 Pj and Sjöberg Stiftelsen: 2023-01-03:3 and the Swedish Research Council:2022-00801.

## Data statement

Study data are available upon request if in line with ethical and legal permissions

## CRediT authorship contribution statement

**Ingrid Glimelius:** Conceptualization, Data curation, Funding acquisition, Investigation, Resources, Writing – original draft, Writing – review & editing. **Sara Ekberg:** Data curation, Formal analysis, Funding acquisition, Investigation, Methodology, Resources, Writing – original draft, Writing – review & editing. **Karin Ekström Smedby:** Funding acquisition, Investigation, Methodology, Resources, Writing – original draft, Writing – review & editing. **Tove Wästerlid:** Conceptualization, Data curation, Funding acquisition, Investigation, Methodology, Project administration, Resources, Writing – original draft, Writing – review & editing.
